# Identify the co-expressed genes of hypertensive nephropathy and diabetic nephropathy

**DOI:** 10.1038/s41598-025-04679-w

**Published:** 2025-06-03

**Authors:** Yanmin Wang, Yiming Wang, Jing Jin, Bing Ma

**Affiliations:** 1 Institute of Statistics and Applied Mathematics, Anhui University of Finance and Economics, Bengbu, China; 2 School of Accountancy, Anhui University of Finance and Economics, Bengbu, China; 3 Bengbu Third People’s Hospital attached to Bengbu Medical University, Bengbu, China

**Keywords:** Hypertension nephropathy, Diabetic nephropathy, Common differentially expressed genes, Protein-protein interaction, Molecular biology, Nephrology

## Abstract

This study focused on the intricate connections between hypertension nephropathy (HN) and diabetic nephropathy (DN) in terms of molecular and pathological mechanisms. The samples were from the Gene Expression Omnibus (GEO) database. GSE37460 and GSE142153 are training sets, and GSE37455 and GSE30529 are validation sets. We found 42 shared differentially expressed genes (DEGs) by means of the differential analysis. The GO/KEGG and GSEA analysis mainly highlights the signal transduction pathways related to the proteasome and cytokines. The eight hub genes identified through the Protein-Protein Interaction (PPI) network analysis include NR4A1, TNFSF10, CX3CR1, EGF, THBD, CXCR4, CCL5, and ATF3. Single-cell sequencing analysis revealed that TNFSF10 and NR4A1 were the most highly expressed in the cells of both HN and DN. Furthermore, five significant microRNAs identified include hsa-miR-1248, hsa-miR-200b-5p, hsa-miR-23b-5p, hsa-miR-3059-5p, and hsa-miR-3065-3p. Six essential transcription factors (TFs) (NFIL3, STAT3, NFKB1, USF1, USF2, and EGR1), 11 important drug chemicals (Cisplatin, Cyclosporine, perfluorooctanoic acid, Quercetin, Tretinoin, bisphenol A, Curcumin, Valproic Acid, Particulate Matter, Simvastatin, and Cadmium), seven related diseases (Atherosclerosis, Glioblastoma, Pulmonary Fibrosis, Asthma, Hepatitis B, Hepatitis C, and Diabetes Mellitus), and ten important RNA-binding proteins (RBPs) (CHTOP, EIF4E, HNRNPK, IGF2BP3, YTHDF3, HNRNPA2B1, RBM47, YBX1, RBFOX2, and RBM10). Finally, molecular docking simulations suggest that Tretinoin and Curcumin may have potential therapeutic value for both HN and DN. This study provides novel therapeutic targets for the combined diagnosis and treatment of HN and DN.

**Supplementary Information:**

The online version contains supplementary material available at 10.1038/s41598-025-04679-w.

## Introduction

Hypertension and diabetes were the common comorbidities (respectively 69.7 and 48.5%)^1^. Two-thirds of patients with type 2 diabetes mellitus (T2DM) have arterial hypertension^2^. Hypertension is twice as frequent in patients with diabetes compared with those who do not have diabetes^3^. Both hypertension and diabetes can result in renal failure. Hypertension is a pivotal risk factor for diabetic nephropathy^4^. Hypertensive nephropathy (HN) and diabetic nephropathy (DN) are the top two common causes of end-stage renal disease (ESRD)^5^. Approximately 1 in 3 adults with diabetes and 1 in 5 adults with high blood pressure may have chronic kidney disease (CKD)^6^. The growing burden of chronic kidney disease (CKD) places substantial financial pressure on patients, healthcare systems, and society. A micro-simulation modelling study showed that annual direct costs (US$) of diagnosed CKD and kidney replacement therapy (KRT) would increase by 9.3% between 2022 and 2027, from $372.0 billion to $406.7 billion for the 31 countries/regions^7^.

Most research has focused on the coexistence between hypertension and diabetes^8–10^. There are also many studies on the effect of hypertension on DN^11–14^. However, few studies have investigated the effect of diabetes on HN. The scholar realized that Glomerular hypertension is an important factor exacerbating glomerular diseases to end-stage renal diseases, especially in hypertensive and diabetic nephropathy^15^. However, to our knowledge, no study exists on the coexistence between HN and DN. There is an apparent association between Hypertensive nephropathy (HN) and diabetic nephropathy (DN). This was further confirmed by a population-based prospective study from China, which states that hypertension control can reduce the incidence of end-stage kidney failure by 23%^16^.

**Fig. 1:**
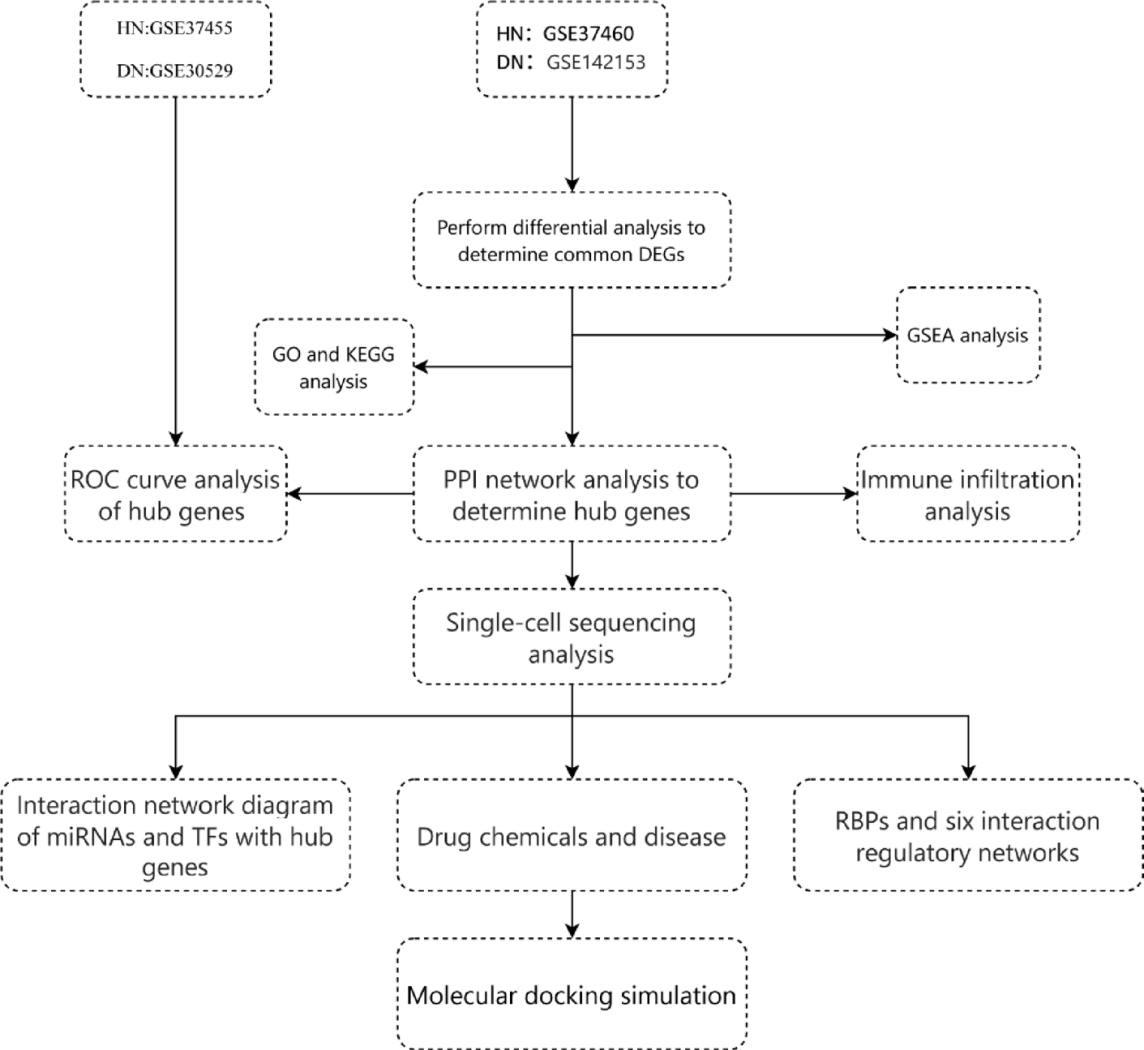
Flow chart of this study.

This study employed four datasets to identify the complex connection between DN and HN. Two of the datasets were used to uncover shared differentially expressed genes (DEGs), followed by Gene Ontology (GO)/Kyoto Encyclopedia of Genes and Genomes (KEGG) and Gene Set Enrichment Analysis (GSEA) analysis. Protein-protein interaction (PPI) was used to analyze shared DEGs and identify hub genes, followed by immune infiltration and diagnostic receiver operating characteristic (ROC) curve analysis. The other two datasets were used to perform ROC curve validation on hub genes. Subsequently, single-cell sequencing analysis was performed to investigate the expression of key genes across different cell types. Based on hub genes, studies were conducted from aspects such as microRNAs (miRNAs), transcription factors (TFs), drug chemical substances, diseases, and RNA-binding proteins (RBPs). A miRNA-lncRNA-mRNA regulatory network was constructed. The inter-regulatory relationships between different types of molecules were intuitively displayed through a combination of the above six network diagrams. Finally, molecular docking simulation experiments were performed to reveal potential therapeutic agents. Figure 1 shows the sequential workflow during the study.

## Materials and methods

### Samples collection

The RNA-Seq data are available in the Gene Expression Omnibus database with the accession numbers GSE37460, GSE37455, GSE142153, GSE30529, GSE174219, and GSE131882. Among these datasets, the first two are used for HN analysis, and the remaining are used for DN analysis. The data set GSE37460, as a training set, was based on the GPL11670 (Affymetrix Human Genome U133 Plus 2.0 Array) and consists of 69 samples: 15 HN patient samples, 27 non-diseased control samples, and 27 IgA nephropathy samples were excluded. The data set GSE37455, as a validation set, was also based on the GPL11670, which was based on the GPL and included 41 samples: 20 HN patient samples and 21 non-diseased control samples. As for DN analysis, the data set GSE142153 (Agilent-014850 Whole Human Genome Microarray 4 × 44 K G4112F) as a training set was based on the GPL6480, which consists of 40 samples: 23 DN patient samples and ten non-diseased control samples, among which 7 ESRD samples were excluded. The data set GSE30529, as a validation set, was based on the GPL571 (Affymetrix Human Genome U133A 2.0 Array) and includes 22 samples of 10 DN patients and 12 non-diseased control participants.

### Shared DEGs between HN and DN

Our research mainly focuses on identifying DEGs in DN and HN training sets. For the DN and HN datasets, the limma package (version 3.62.2) in R is used for differential analysis to determine DEGs. DEGs with a p-value less than 0.05 and |log2FC| > 0.585 are considered statistically significant. We generated Venn diagrams to visualise the intersection situations of DEGs in the two datasets, and volcano plots were drawn to show the DEGs’ situation in each dataset. The genes in the volcano plot are DEGs with a p-value less than 0.001 and |log2FC| > 2. The R package Venndiagram (version 1.7.3) and ggplot2 (version 3.5.2) drew the Venn diagrams and volcano plots, respectively.

### GO, KEGG, and GSEA enrichment analysis

The R package ClusterProfiler (version 4.16.0) was applied to perform the GO function and KEGG signalling pathway enrichment analysis on 42 DEGs^17,18^. The screening conditions for GO analysis are an FDR of less than 0.25 and an adjusted p-value of less than 0.05. The top 10 items of BP, CC, and MF (only two items for CC) were observed in the bubble plot. Furthermore, we used 42 DEGs to generate a circular plot to explore the GO further. The screening condition for KEGG analysis is a P-value less than 0.05, and a bar graph is generated for display.

In our study, we used all genes from the two datasets for GSEA. After obtaining the data, we screened pathways according to an adjusted p-value less than 0.25, a p-value less than 0.05, and an absolute value of the normalised enrichment score (NES) greater than 1. We selected ten critical pathways in each dataset according to the NES value. We generated enrichment and ridge plots for each dataset to present the results visually. R created the enrichment plots and ridge plots.

### PPI and hub genes between HN and DN

We utilised the STRING database (version 12.0, https://cn.string-db.org/) to identify the PPI of the shared DEGs between HN and DN^19^. The database compiles the known and predicted protein connections, including physical and functional aspects, which helps analyse PPI. We set the low confidence level as the minimum interaction score of 0.150 to construct the PPI. Subsequently, Cytoscape (version 3.10.3), a platform-independent software for network analysis and visualization, was applied to present these results and obtain better visualisation effects^20^. When screening hub genes through PPI, the Cytohubba plugins and MCODE of Cytoscape were used. Five algorithms were performed to screen hub genes, including MCODE, MCC, MNC, Degree, and EPC^21,22^. Twelve hub genes were obtained using the MCODE algorithm, and another four algorithms selected the top 10 hub genes. After that, the intersection of the results of the five algorithms was taken to determine the core hub genes, which were listed in a Venn diagram.

### ROC analysis and immune infiltration analysis of hub genes

First, we screened the expression data of core hub genes to generate heat maps of the two datasets and used grouped data to generate two comparison diagrams of HN and DN. Subsequently, four ROC curves were drawn, including the training set and test set of DN and HN. Each figure contains all the core hub genes.

In our analysis, the linear relationship between 22 tumour-infiltrating immune cells (TIICs) and eight-core hub genes and the correlation among immune cells were visualised by the R package linkET (version 0.0.3). The CIBERSORT, an analytical tool from the Alizadeh Lab and Newman Lab, was used to estimate the abundance of 22 TIICs using the gene expression data of the training cohort.

### Single-cell expression levels of key genes

We selected two gene expression datasets: GSE131882 (containing three diabetic nephropathy samples and three normal control samples) and GSE174219 (containing two hypertensive nephropathy samples and one normal control sample) from the GEO database, and performed analyses using the R package Seurat (version 5.3.0) and CellChat (version 1.5.0)^23,24^. We retained genes expressed in more than three cells and cells with gene expression levels between 200 and 4000, while excluding cells with mitochondrial gene expression exceeding 10%. A total of 3000 highly variable genes were set, and the function LogNormalize in the Seurat package was used for data normalization. For single-cell data of hypertensive nephropathy, we retained cells with gene expression levels between 200 and 5000 and excluded cells with mitochondrial gene expression exceeding 20%. In this study, PCA analysis was first used to reduce the dimensionality of the data. Then, the function UMAP in the Seurat package was applied again to further reduce the data dimension, with the dim set to 20. Cells were clustered using the KNN method, with dims set to 20 and the resolution set to 0.2. Cells were annotated based on the characteristic genes of different cell types. The FindMarkers function in the Seurat package was employed to identify differential expression between groups. Finally, the CellChat package was utilized to perform cell-cell communication analysis.

### The MiRNAs, TFs, drug molecules, diseases, and RBPs predicted by hub genes

MiRNA is a naturally occurring small non-coding RNA that affects protein expression by binding to gene transcripts^25^. TFs hold great significance in regulating transcription rate and binding to specific genes, which can provide valuable molecular insights^26^. To identify putative Hub gene-miRNAs, we used the miRWalk database (version 3.0 at http://mirwalk.umm.uni-heidelberg.de/) to sift through pairs with a quantity exceeding 20. Moreover, the miRDB database (version 6.0 at https://mirdb.org/) was applied in this screening procedure. The Hub gene-miRNAs were obtained by overlapping the genes identified in both databases. After that, visualisation was carried out using Cytoscape. ENCORI (https://rnasysu.com/encori/*)* was used to predict interactions between miRNAs and lncRNAs, with lncRNAs having more than one CLIP-seq dataset retained. The lncRNA-mRNA-miRNA regulatory network was visualized by Cytoscape. For Hub gene-TF, we utilised the networkanalyst database (https://www.networkanalyst.ca/) to obtain the results. Subsequently, a network diagram was generated by Cytoscape.

We utilised the networkanalyst database and the Comparative Toxicogenomics Database (CTD: https://ctdbase.org/) to predict the interactions between hub genes and drugs. Filter the small molecule structures of eight hub genes using the criterion that the reference count is greater than 2. Determine the key related chemicals by taking the intersection of the results obtained from the two datasets. After that, the interactions between pharmaceutical chemicals and hub genes were imported into Cytoscape for visualisation. Moreover, we explored the connections between hub genes and diseases to identify related diseases that share common hub genes. At first, we employed the DisGeNET database (version 7.0, http://www.disgenet.org/) and the MalaCards database (version 5.17, https://www.malacards.org/) to screen for relevant genes. Then, the expected results extracted from comparing the two databases were imported into Cytoscape to establish the relationship between hub genes and diseases.

We used the ENCORI database (https://rnasysu.com/encori/) to identify central genes-RBPs. Only the genes with more than one CLIP-seq experiment were selected. Then, the filtered hub genes-RBPs were imported into the Cytoscape to generate a network diagram. By considering eight hub genes, five predicted significant miRNAs, six predicted TFs, eleven predicted pharmaceutical chemicals, seven predicted diseases, and ten predicted RBPs, we generated a network diagram that demonstrates the regulatory relationships among different networks.

### Identification of potential therapeutic drugs

To explore potential therapeutic drugs or compounds for HN and DN, we conducted molecular docking simulations using AutoDockTools (version 1.5.7), a suite of automated docking tools, to predict the interaction patterns between protein active sites and ligands. The protein structures of these hub genes and drugs were obtained from the RCSB Protein Data Bank (https://www.rcsb.org/) and PubChem (https://pubchem.ncbi.nlm.nih.gov/*).* Finally, PyMOL (version: 3.0.0), a molecular visualization system, was used to visualize the spatial 3D structures.

### Statistical analysis

This statistical analysis and visualisation were done using R (version 4.4.0, Windows 64-bit). The Wilcoxon rank-sum test was adopted, and grouping comparison diagrams were drawn to analyse hub genes. In addition, Spearman’s correlation coefficient was used to evaluate the relationship between immune cells. The levels of statistical significance were defined as *p* < 0.05 (*), *p* < 0.01 (**), and *p* < 0.001 (***), while “ns” indicates no significant difference.

## Result

### Identification of DEGs shared by HN and DN

**Fig. 2:**
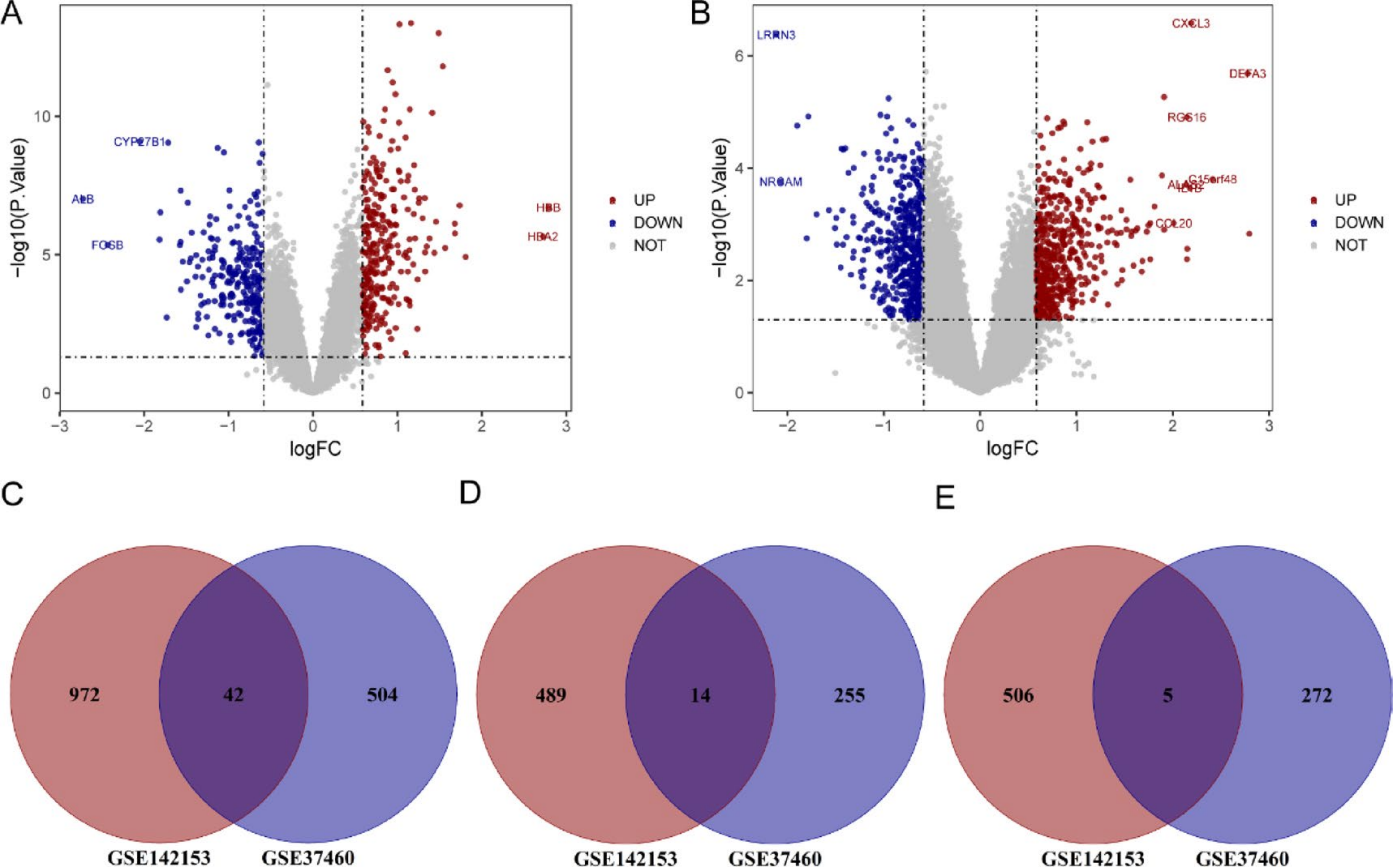
Volcano plots and Venn diagrams. (**A**, **B**) Volcano plot of HN and DN. (**C**) The intersection of all DEGs in two diseases. (**D**, **E**) The junction of up-regulated DEGs and down-regulated DEGs in two diseases.

In the HN dataset GSE37460, 546 genes showing differential expression were detected, including 269 up-regulated and 277 down-regulated genes. Similarly, in the DN dataset GSE142153, 1014 DEGs (503 up-regulated and 511 down-regulated) were found. Figure 2 shows two volcano plots, with Fig. 2A (HN) and Fig. 2B (DN). Red and blue dots represent the up-regulated and down-regulated genes, respectively. Meanwhile, the gene names with p-values less than 0.001 and | log2FC| > 2 are displayed. We generated Venn diagrams to determine 42 shared differentially expressed genes between HN and DN (Fig. 2C). Among these genes, 14 were up-regulated DEGs (Fig. 2D) and five were down-regulated DEGs (Fig. 2E). The 42 DEGs are given in Supplementary Table S1.

### GO, KEGG, and GSEA

**Fig. 3:**
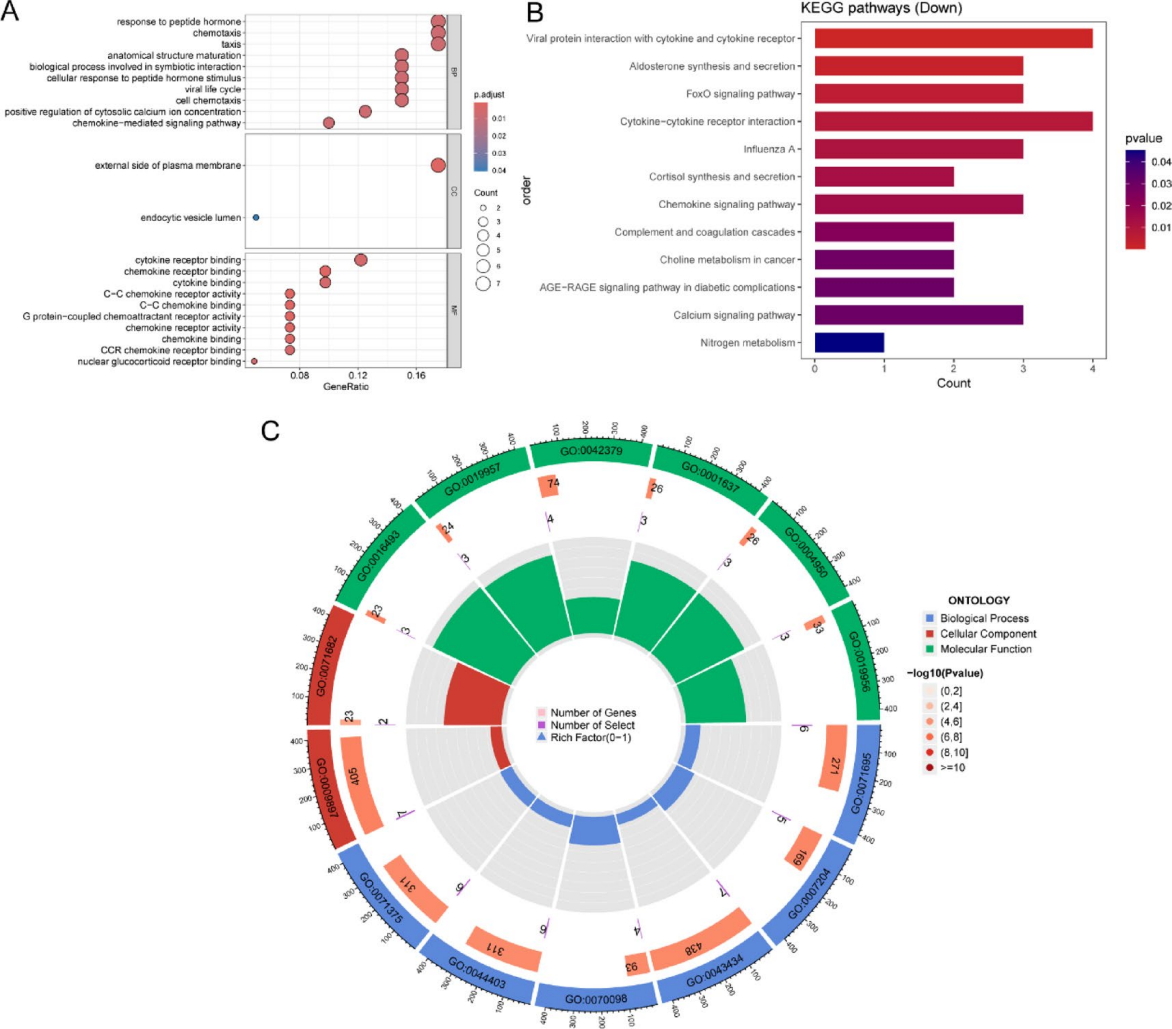
GO and KEGG analysis of 42 DEGs. (**A**) Bubble chart of GO enrichment. (**B**) Bar chart of KEGG. (**C**) Circular diagram of GO enrichment.

Figure 3A shows the GO analysis of the top 10 items in the BP, CC, and MF categories, among which there are only two in the CC category. DEGs show significant enrichment, including response to peptide hormones, chemotaxis, and taxis in the BP category. The highly enriched external side of the plasma membrane is in the CC category, and cytokine receptor binding and chemokine receptor binding are in the MF category. KEGG enrichment indicates Aldosterone synthesis, secretion, and the FoxO signalling pathway. In addition, KEGG enrichment also shows the AGE − RAGE signalling pathway in diabetic complications (Fig. 3B). A circular diagram is generated to show the GO enrichment of 42 DEGs (Fig. 3C).

**Fig. 4:**
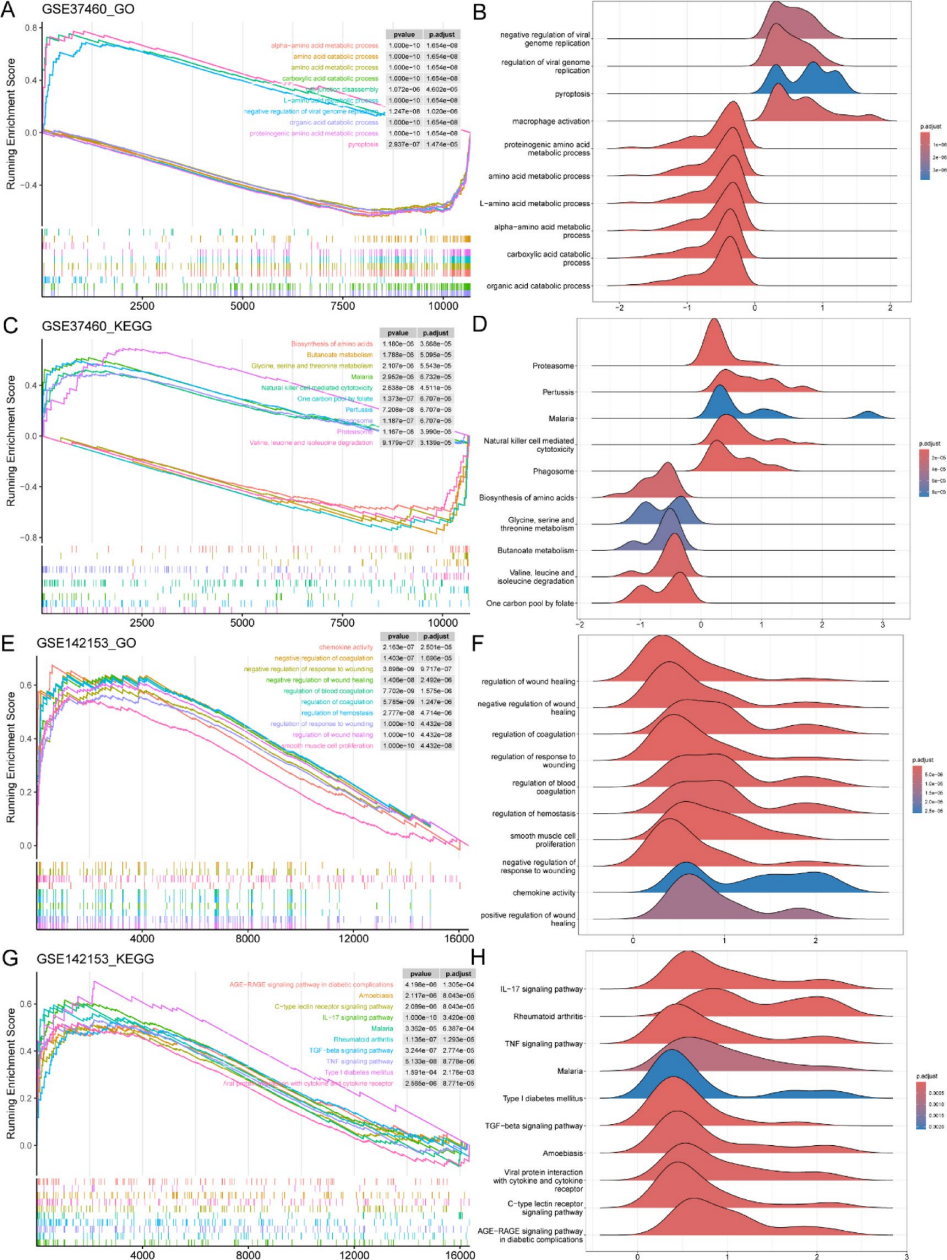
GSEA functional enrichment analysis. (**A**) Classic diagrams of 10 pathways of HN in the GO database. (**B**) Ridge plots of 10 pathways of HN in the GO database. (**C**) Classic diagrams of 10 pathways of HN in the KEGG database. (**D**) Ridge plots of 10 pathways of HN in the KEGG database. (**E**) Classic diagrams of 10 pathways of DN in the GO database. (**F**) Ridge plots of 10 pathways of DN in the GO database. (**G**) Classic diagrams of 10 pathways of DN in the KEGG database. (**H**) Ridge plots of 10 pathways of DN in the KEGG database.

According to the GO and KEGG databases, GSEA was performed on all HN genes, and ten essential pathways were obtained. The GO database shows four up-regulated pathways and six down-regulated pathways. The up-regulated pathways include macrophage activation. The down-regulated pathways include the amino acid metabolic process (Fig. 4A). Figure 4B shows the mountain plots of these ten pathways in HN. The KEGG database shows five up-regulated pathways and five down-regulated pathways. The up-regulated pathways include proteasome, pertussis, malaria, natural killer cell-mediated cytotoxicity, and phagosome. The down-regulated pathways include the biosynthesis of amino acids and butanoate metabolism (Fig. 4C). A ridge plot is generated to show these ten pathways (Fig. 4D). Similarly, DN’s ten most essential pathways are revealed, and all of these pathways are up-regulated. The GO database includes positive regulation of wound healing, chemokine activity, smooth muscle cell proliferation, regulation of blood coagulation, regulation of coagulation, and regulation of wound healing (Fig. 4E). The KEGG database includes the AGE-RAGE signalling pathway in diabetic complications, amoebiasis, type I diabetes mellitus, and malaria (Fig. 4G). A mountain plot is generated to describe the respective ten pathways in DN (Fig. 4F,H).

### PPI screened hub genes of HN and DN

**Fig. 5:**
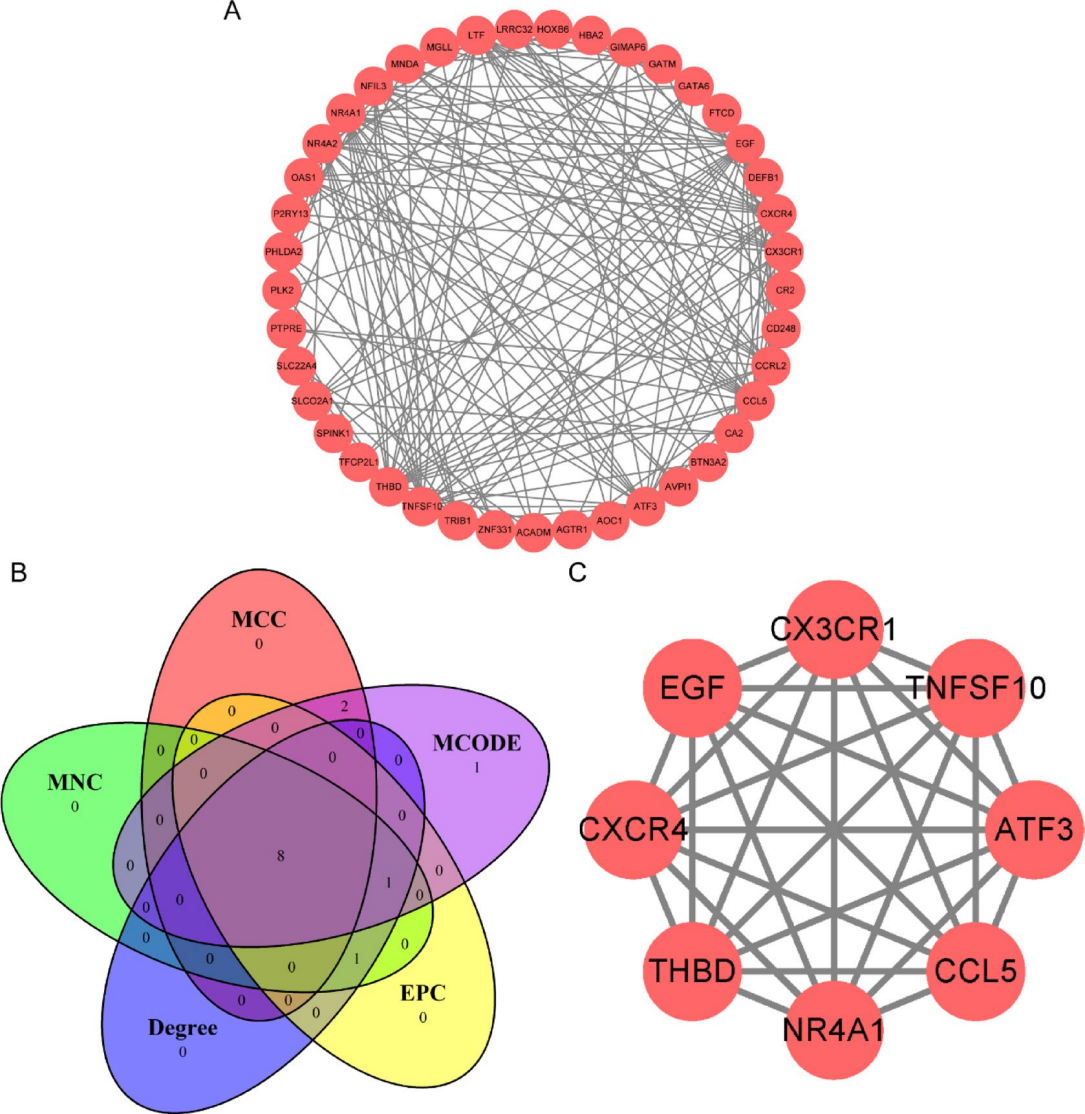
PPI and hub gene. (**A**) Shared DEGs of HN and DN in PPI (41 nodes,186 edges). (**B**) Venn diagram of hub genes screened from five algorithms: MCODE, MCC, MNC, Degree, and EPC. (**C**) Network diagram of eight hub genes (8 nodes, 28 edges).

To study the interaction of 42 DEGs, the shared DEGs were imported into the STRING database for analysis, and a PPI network diagram including 41 nodes and 186 edges was obtained (Fig. 5A). Subsequently, the Cytoscape plugin in Cytohubba was used to identify hub genes. The MCODE algorithm yielded 12 hub genes. The remaining four algorithms retained the top ten hub genes. Finally, eight hub genes were obtained based on the results of the five algorithms, as shown in Fig. 5B. The eight hub genes are NR4A1, TNFSF10, CX3CR1, EGF, THBD, CXCR4, CCL5, and ATF3 (Fig. 5C).

### ROC and immune infiltration analysis of 8 hub genes

**Fig. 6:**
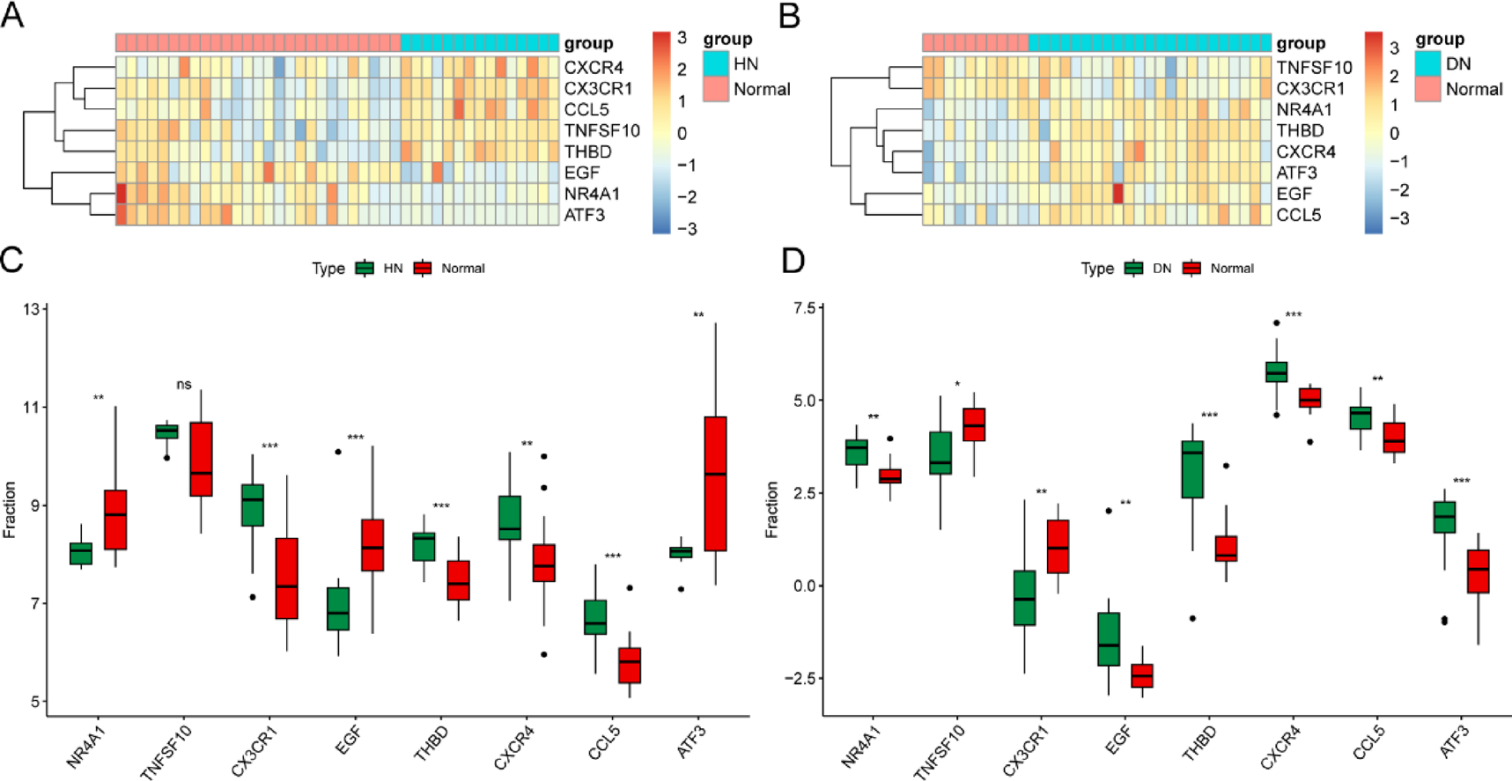
Heat map and group comparison diagram of hub genes. (**A**, **B**) Heat map of eight hub genes in HN and DN. (**C**, **D**) Group comparison diagram of eight hub genes in HN and DN.

The two heat maps presented can visualise the comprehensive expression patterns of eight hub genes in HN (Fig. 6A) and DN (Fig. 6B). Our analysis involves examining eight hub genes and comparing the differences between the disease groups of HN and DN and their respective healthy groups. Figure 6C shows the control results in HN and finds no significant difference in TNFSF10. The remaining seven genes all show substantial differences. In the test of DN, all the hub genes showed significant differences between the two groups (Fig. 6D).

**Fig. 7:**
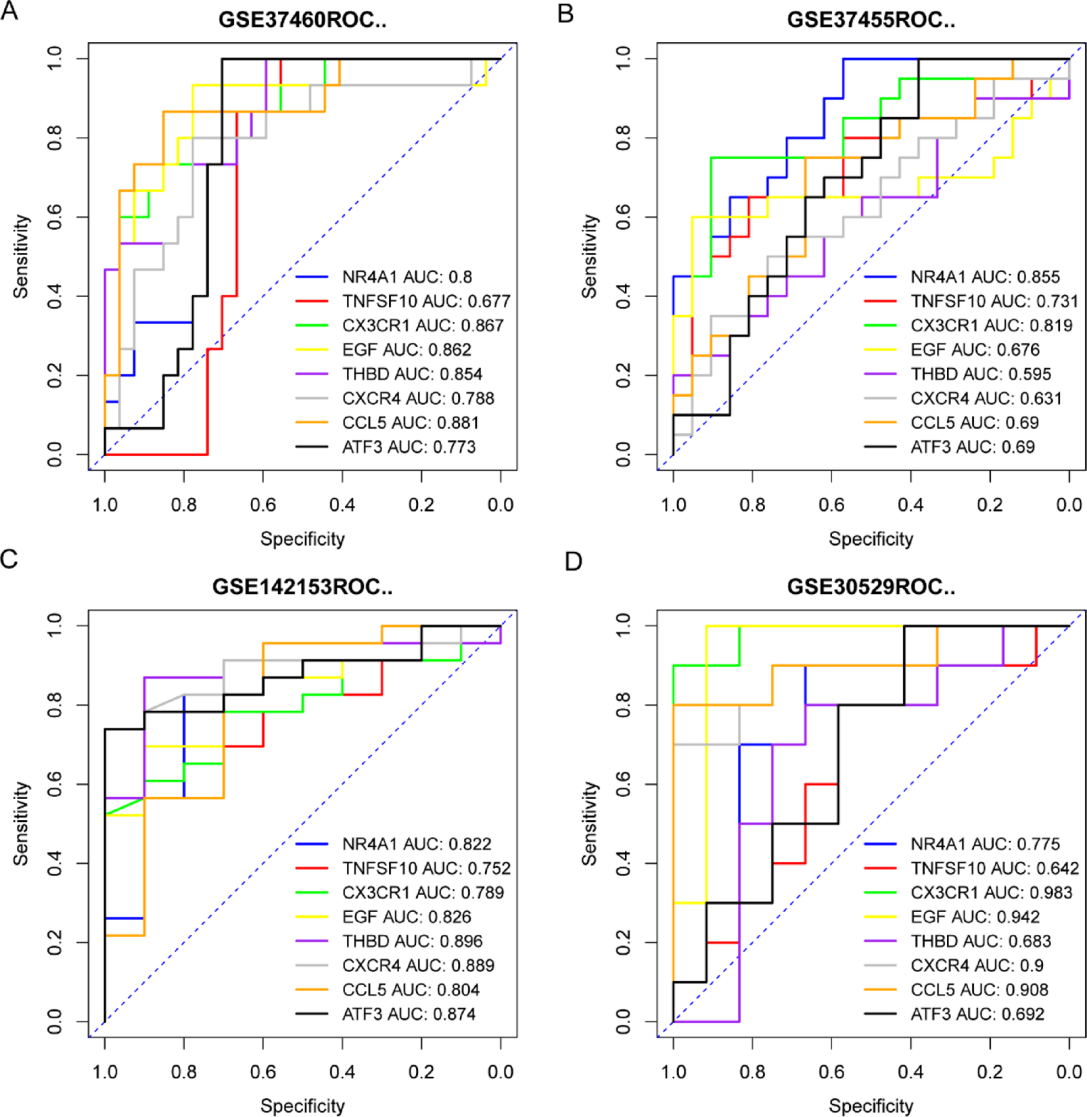
ROC curves of eight hub genes in HN and DN. (**A**, **B**) ROC curve of HN training set and validation set. (**C**, **D**) ROC curve of DN training set and validation set.

The diagnostic effectiveness of eight hub genes for HN and DN was evaluated by diagnostic ROC curve analysis and further verified by including a validation set. Figure 7 shows the ROC analysis results of eight hub genes. The ROC analysis results of HN include the training set (Fig. 7A) and the validation set (Fig. 7B). Most hub genes show an AUC greater than or close to 0.7, and a few have an AUC greater than 0.8. Specifically, in the training set, the genes with an AUC greater than 0.8 are NR4A1, CX3CR1, EGF, THBD, and CCL5; in the test set, they include NR4A1 and CX3CR1. Similarly, Fig. 7C,D display the ROC curves of DN’s training and testing cohorts. In the training cohort, except for the AUC of NR4A1 and TNFSF10, the AUC of the remaining genes is all greater than 0.8; in the validation set, the genes with an AUC greater than 0.8 include CX3CR1, EGF, CXCR4, and CCL5.

**Fig. 8:**
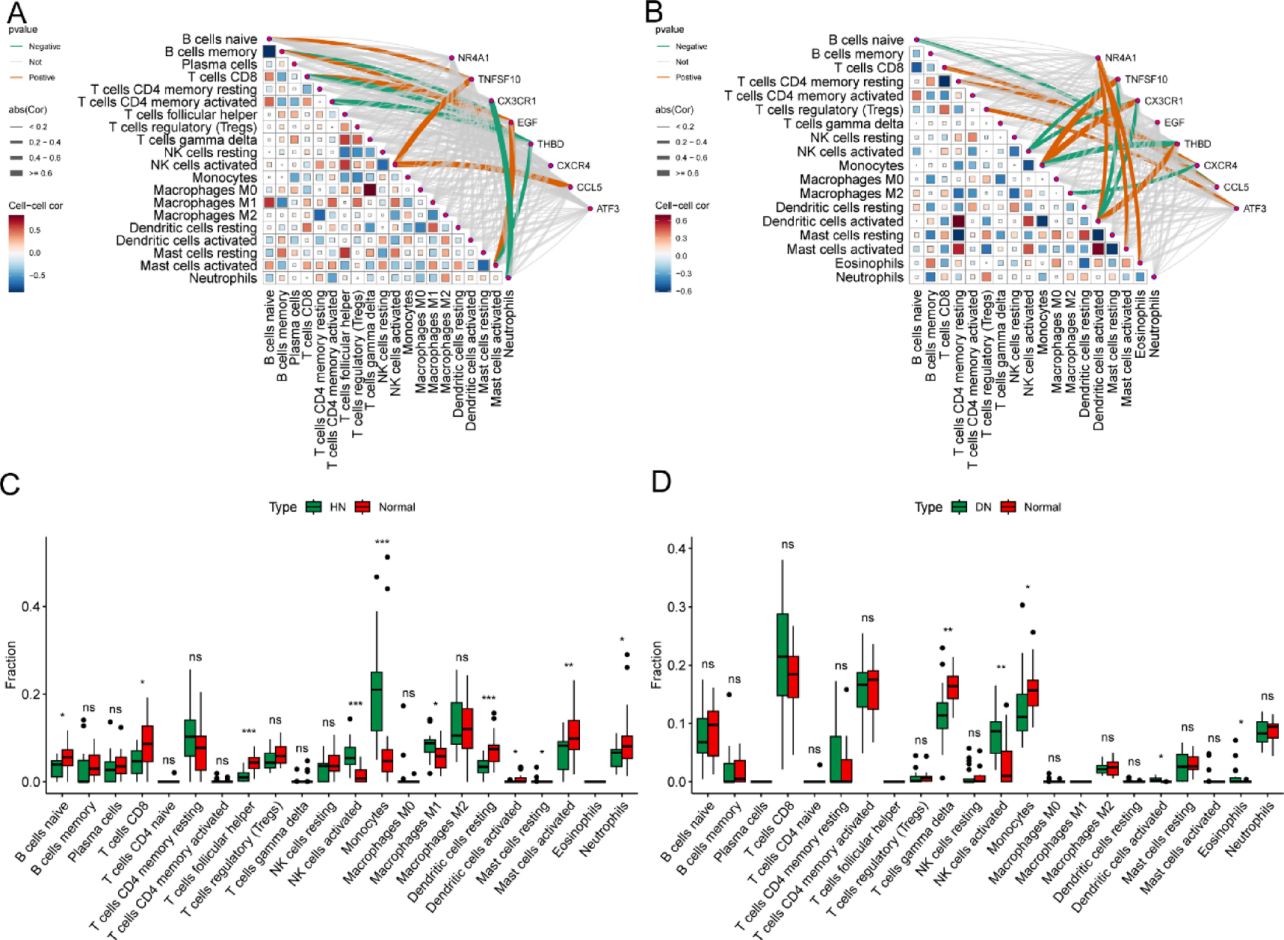
Analysis of immune cell infiltration. (**A**, **B**) Correlation between hub genes and immune cell infiltration in HN and DN. (**C**, **D**) Differential analysis of immune cell infiltration in HN and DN.

Figure 8 shows the correlation between eight hub genes and 22 types of immune infiltrating cells and uses the CIBERSORT algorithm to evaluate the difference in abundance of 22 types of immune cell types between diseased and non-diseased samples in HN and DN. Specifically, Fig. 8A,B illustrate the correlations between eight hub genes and 22 kinds of immune cells in HN and DN. The red lines indicate a positive correlation between the genes and the immune cells, while the green lines represent a negative correlation. There were differences in the abundance of 11 types of immune cells between the normal group and the diseased group in HN (Fig. 8C). In DN (Fig. 8D), the expressions of five immune cells show significant differences. In HN and DN, the immune cells with substantial differences in common were activated natural killer cells, monocytes, and activated dendritic cells.

### Expression of key genes across different cell types

**Fig. 9:**
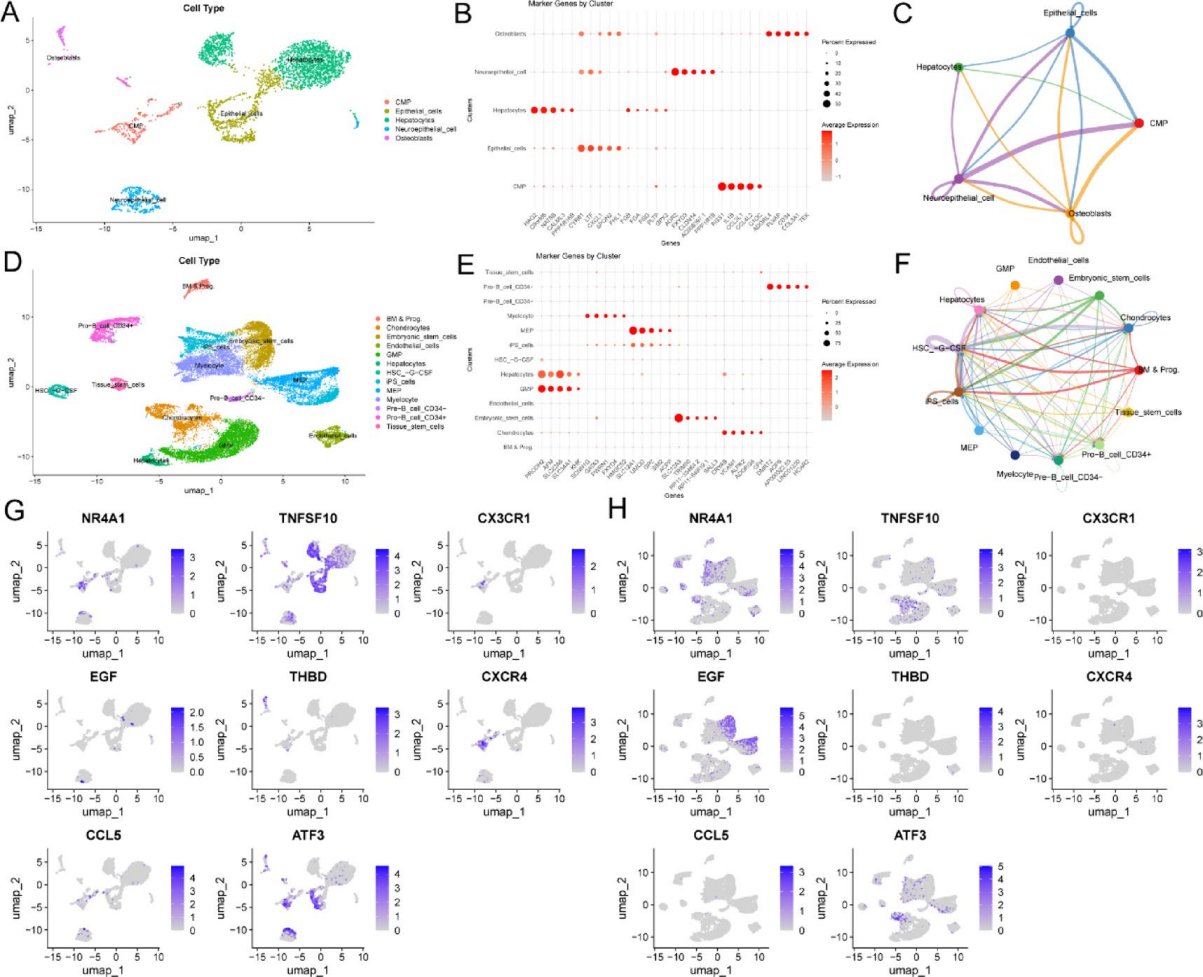
Single-cell sequencing analysis of HN and DN. (**A**, **D**) UMAP plots of HN and DN. (**B**, **E**) Dot plots showing the expression of 30 marker genes across different cell types in HN and DN. (**C**, **F**) Cell communication diagrams between different cell types in HN and DN. (**G**, **H**) Graphs displaying the expression of 8 key genes across different cell types in HN and DN.

Through Fig. 9A,D, UMAP clustered HN and DN cells into 5 and 13 different clusters, respectively. Dot plots displayed the expression of 30 marker genes in HN and DN across different cell types. The color and size of the dots reflect gene expression levels, from which we can observe in which cell types specific genes are highly expressed, aiding in the screening of key cell markers related to the disease (Fig. 9B,E). Figure 9C,F show the signal communication networks between different cell types in HN and DN. Finally, the expression of 8 key genes varied across different cell types. In HN single-cell data, TNFSF10 and ATF3 were highly expressed in hepatocytes and epithelial cells; in DN single-cell data, NR4A1 and EGF showed the highest expression levels (Fig. 9G,H).

### The MiRNAs, TFs, drug molecules, diseases, and RBPs predicted by hub genes

**Fig. 10:**
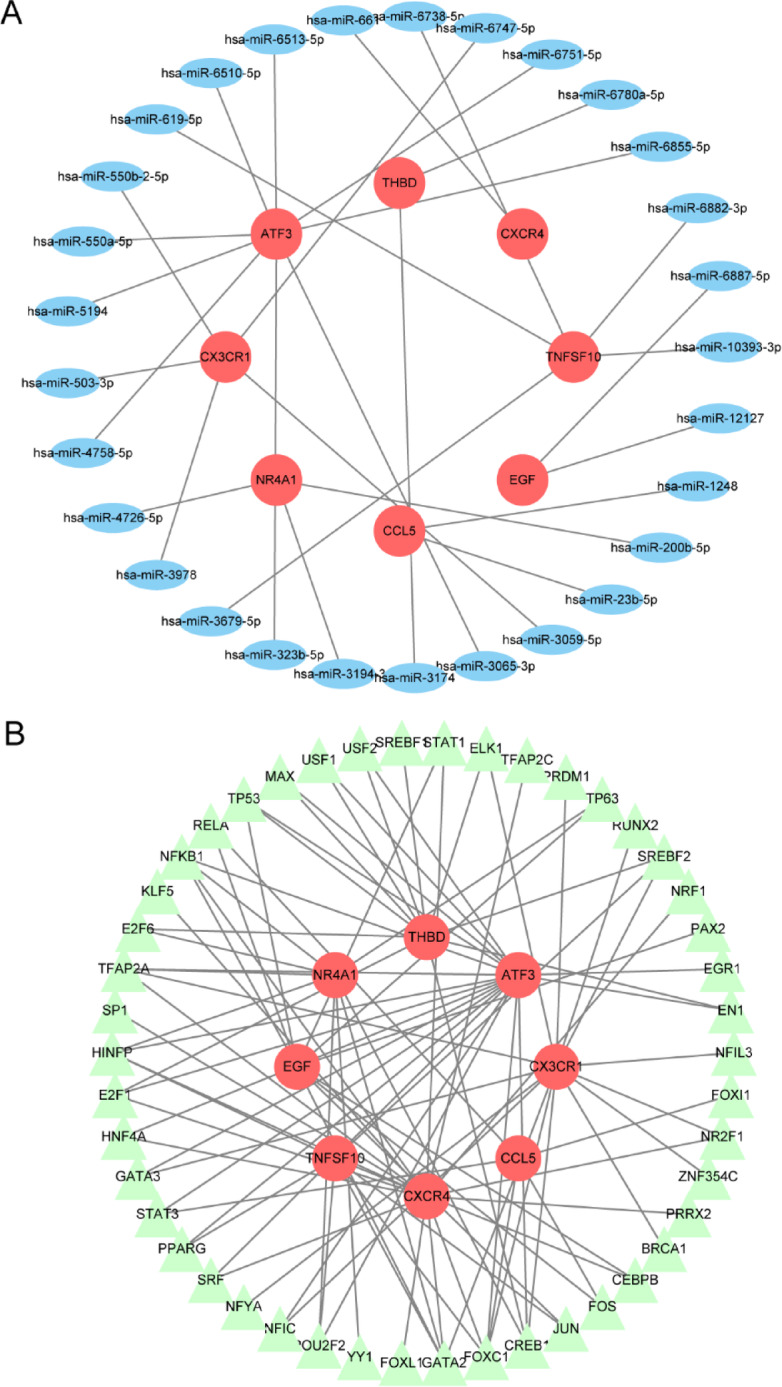
The mutual regulatory network. (**A**) Hub genes-miRNAs. Red circular nodes represent hub genes, and blue oval nodes represent miRNAs (37 nodes and 29 edges). (**B**) Hub genes-TFs. Green triangular nodes represent TFs (54 nodes and 93 edges).

**Fig. 11:**
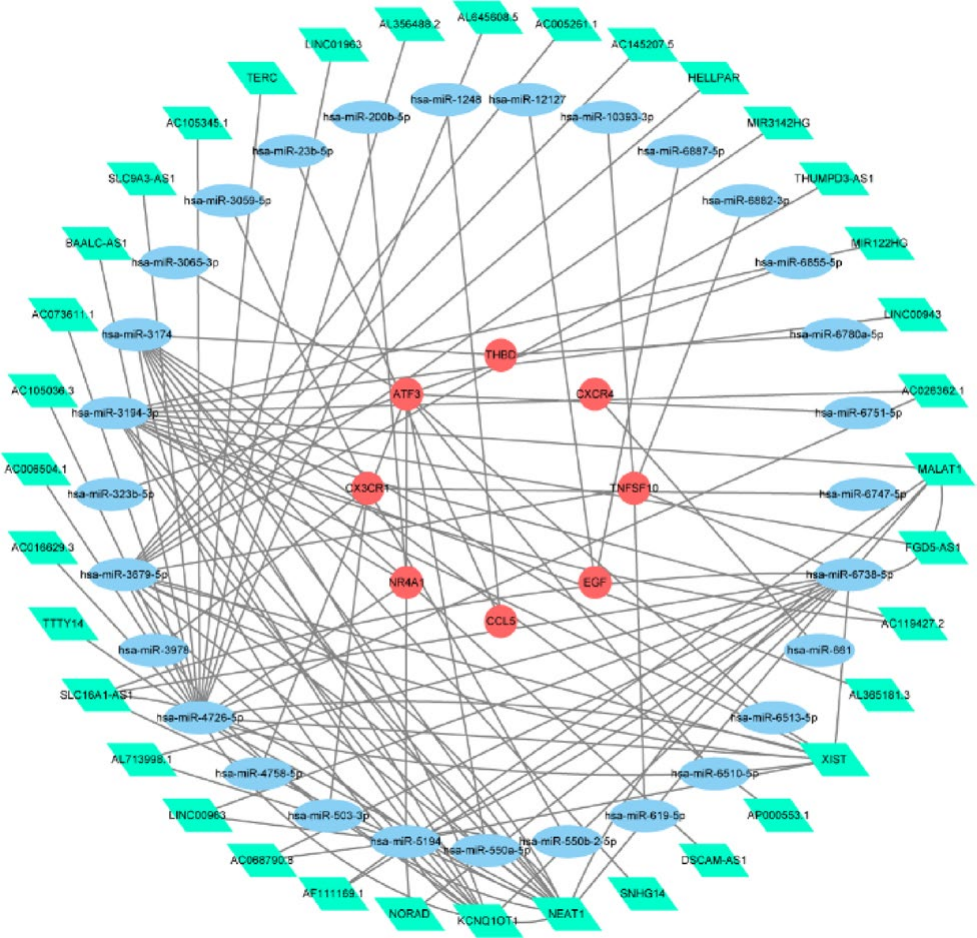
The miRNA-lncRNA-mRNA regulatory network constructed from 8 key genes (73 nodes, 109 edges). Green parallelograms represent lncRNAs.

The Mirwalk database identified 130 hub gene-miRNA interactions through screening. In addition, 634 miRNAs that interact with hub genes were screened from the miRDB database. By taking the intersection of the two databases, 29 hub gene-miRNA interactions were obtained. Importantly, these interactions involve all eight hub genes: NR4A1, TNFSF10, CX3CR1, EGF, THBD, CXCR4, CCL5, and ATF3. Figure 10A shows the hub gene-miRNA interaction. In this network, five significant miRNAs are at least one of hsa-miR-1248, hsa-miR-200b-5p, hsa-miR-23b-5p, hsa-miR-3059-5p, and hsa-miR-3065-3p. Subsequently, we searched the network analysis database and identified 46 TFs that interact with eight hub genes. Figure 10B shows the interaction network diagram between hub genes and TFs. Moreover, NFIL3, STAT3, NFKB1, USF1, USF2, and EGR1 have been identified as six crucial transcription factors. The miRNA-lncRNA-mRNA regulatory network shows that long non-coding RNAs (lncRNAs) KCNQ1OT1 and NEAT1 are closely associated with miRNAs, suggesting that they may act as ceRNAs (competing endogenous RNAs). By binding to miRNAs, they influence the regulatory effects of miRNAs on mRNAs (Fig. 11).

**Fig. 12:**
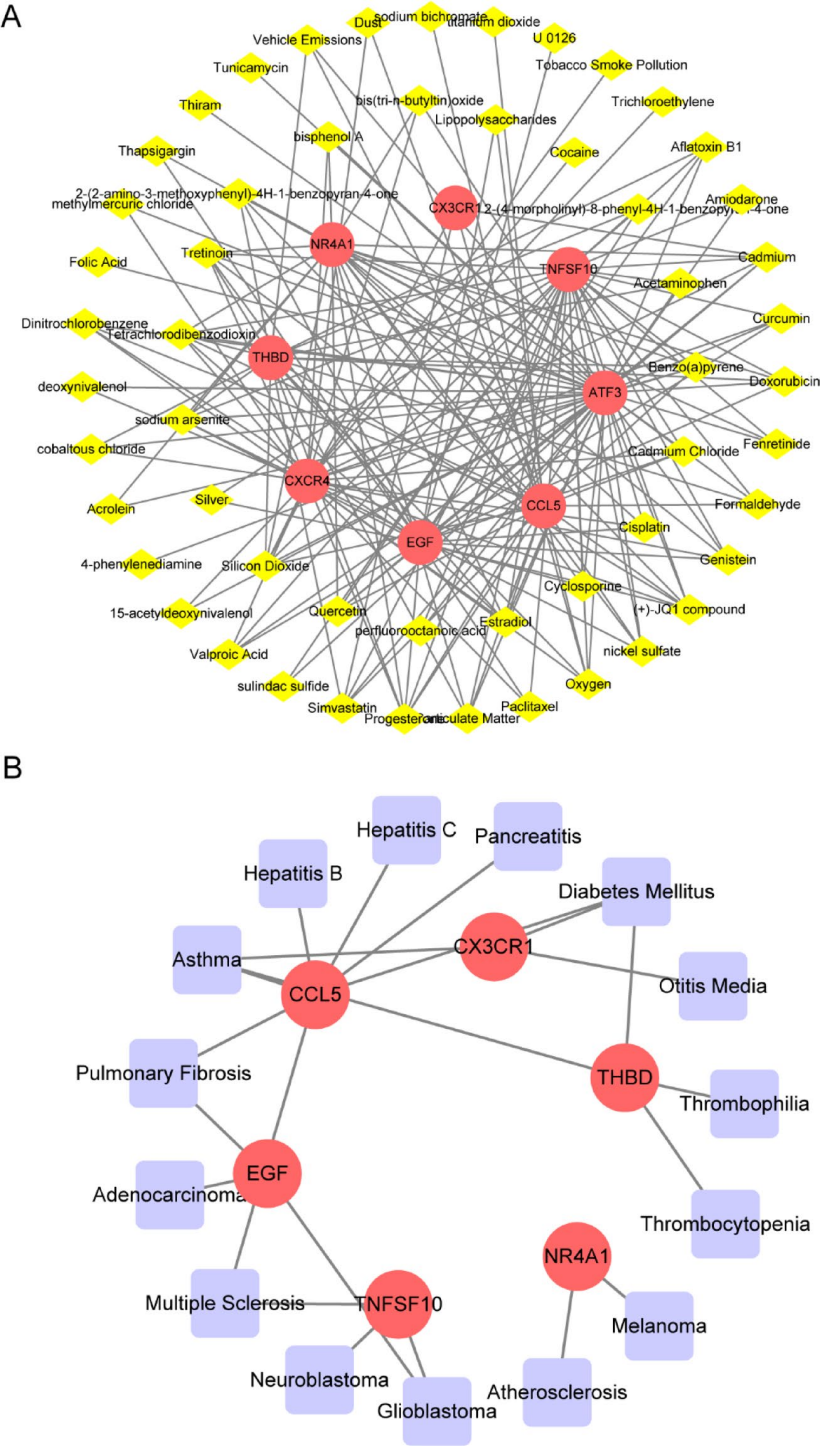
Hub gene-drug chemical and hub gene-disease interaction regulatory network. (**A**) Hub gene-drug chemical. Yellow diamond nodes represent drug chemical substances (67 nodes and 201 edges). (**B**) Interaction network diagram of six hub genes and diseases. Purple square nodes represent diseases (21 nodes and 22 edges).

The network analysis database identified 1064 drug chemicals that interact with hub genes. At the same time, screening the CTD revealed 83 hub gene-drug chemical interactions. The intersection of these two databases yielded 59 chemical substances. Importing these into Cytoscape reveals the interaction network of 8 hub genes and 59 drug chemicals. Figure 12A shows 11 necessary drug chemicals such as cisplatin, cyclosporine, perfluorooctanoic acid, quercetin, tretinoin, bisphenol A, curcumin, valproic acid, particulate matter, simvastatin, and cadmium. Similarly, we screened the DisGeNET and MalaCards databases and identified 217 and 7468 hub gene-disease names, respectively. The overlap of these two databases yielded 15 diseases related to hub genes, and they were visualised through Cytoscape. The network reveals the participation of 6 hub genes and 15 diseases (Fig. 12B). Examining gene-disease correlations emphasises that seven diseases, such as atherosclerosis, glioblastoma, pulmonary fibrosis, asthma, hepatitis B, hepatitis C, and diabetes mellitus, have essential associations with HN and DN.

**Fig. 13:**
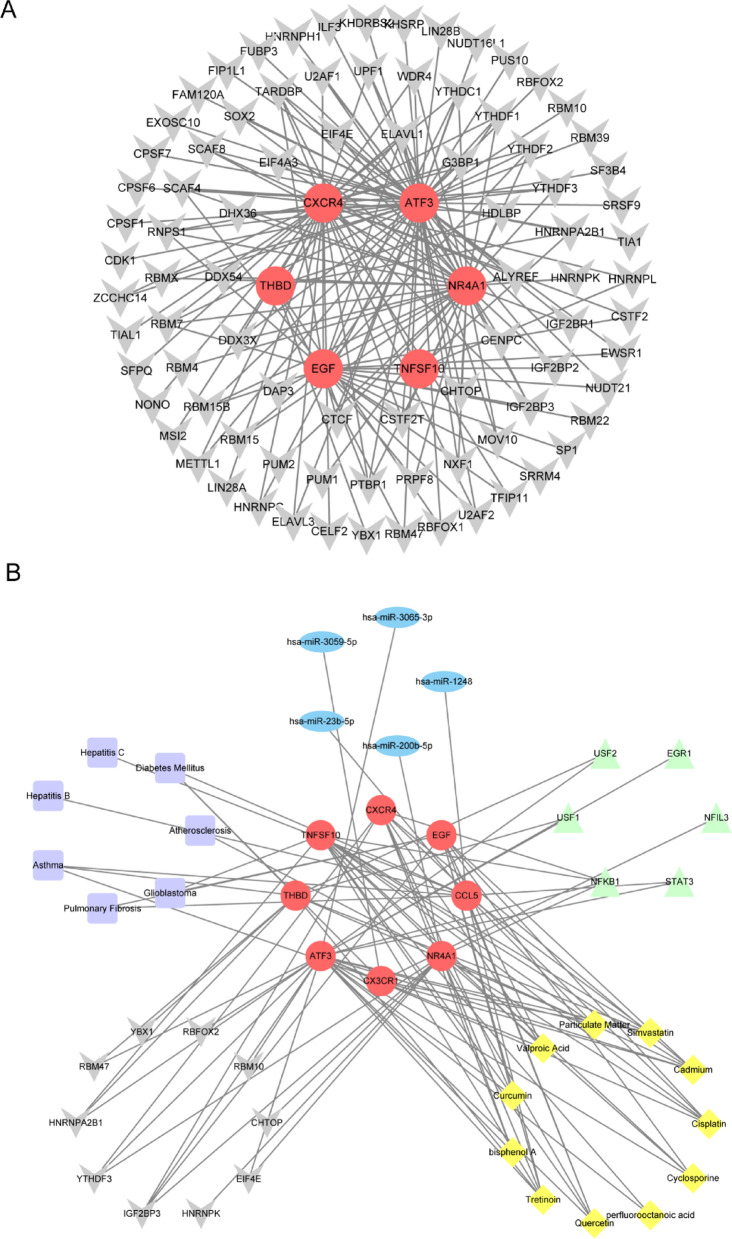
Hub gene-RBPs network diagram and the combined regulation of six kinds of network diagrams. (**A**) Hub gene-RBPs. Grey “V”-shaped nodes represent RBPs (91 nodes, 171 edges). (**B**) The complex interrelationships among Hub genes, miRNAs, TFs, pharmaceutical chemicals, diseases, and RBPs (47 nodes, 100 edges).

We input the 85 RBPs related to hub genes, which were screened from the starBase database, into Cytoscape. The visualisation results (Fig. 13A) reveal the association between 6 hub genes and 85 RBPs, indicating common characteristics between these RBPs and HN and DN. The ten most essential RBPs were determined to be CHTOP, EIF4E, HNRNPK, IGF2BP3, YTHDF3, HNRNPA2B1, RBM47, YBX1, RBFOX2, and RBM10. Finally, Fig. 13B shows the network diagram of the mutual regulatory relationship of these six molecules. Among them, hsa-miR-1248, hsa-miR-23b-5p, STAT3, Quercetin, Tretinoin, Particulate Matter, Simvastatin, Asthma, Hepatitis B, Hepatitis C, Diabetes Mellitus, and Pulmonary Fibrosis have an interaction relationship centred on CCL5. Similarly, hsa-miR-3059-5p, Asthma, Diabetes Mellitus, NFIL3, Simvastatin, and Cadmium have an interaction relationship centred on CX3CR1. At the same time, Valproic Acid, Cadmium, Pulmonary Fibrosis, Glioblastoma, IGF2BP3, Curcumin, Cisplatin, Cyclosporine, Quercetin, and Tretinoin show an interaction relationship centred on EGF. Notably, the interaction relationship centred on ATF3 is as high as 25. The interaction relationship tables of the above six different types of molecular complexes can be found in Supplementary Table S2.

#### Molecular docking simulation

**Fig. 14:**
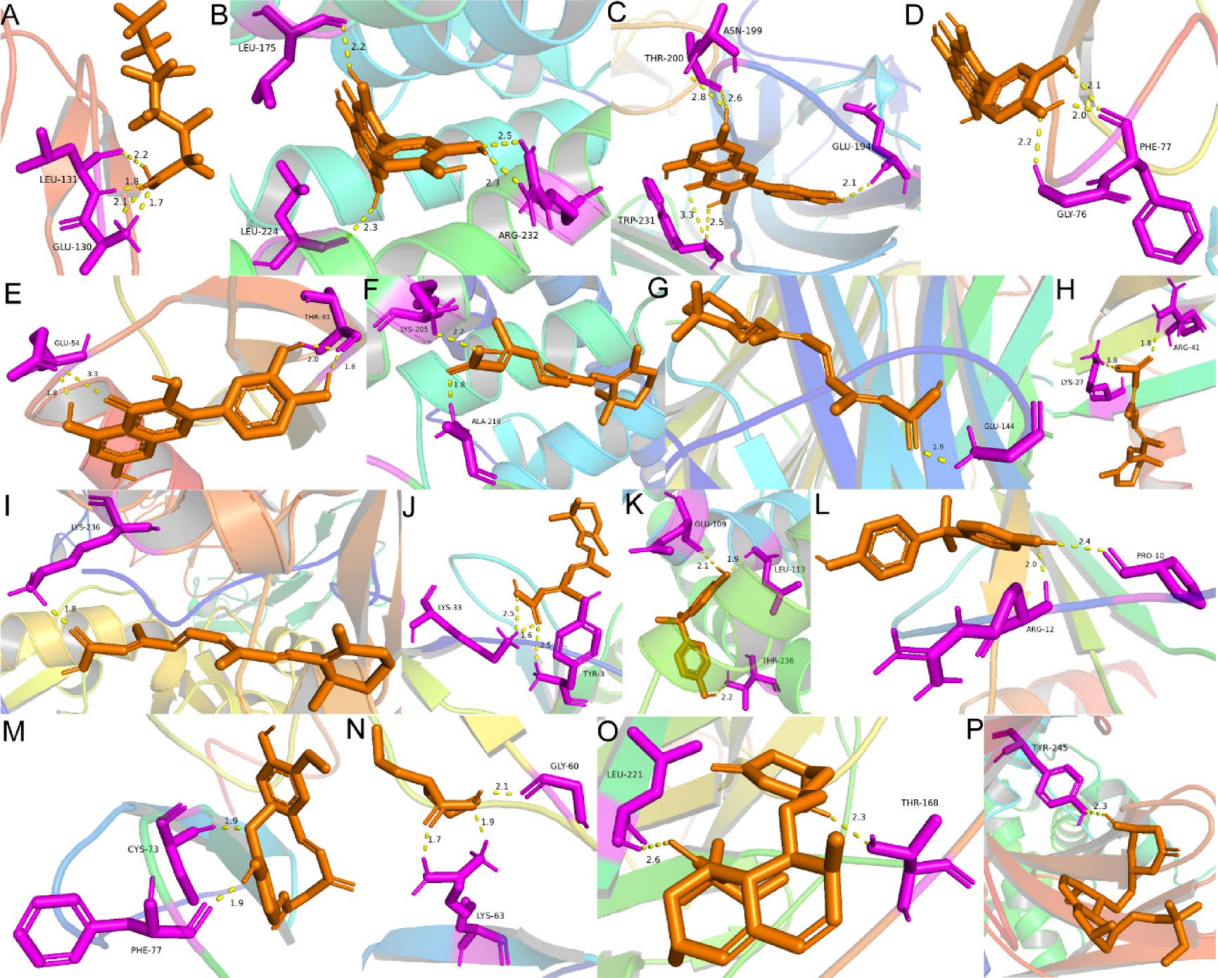
Results of molecular docking simulation by PyMOL (version: 3.0.0). (**A**) Schematic diagram of the molecular structures of perfluorooctanoic acid and TNFSF10, where yellow dashed lines indicate the presence of hydrogen bonds, and the numbers beside them represent the distances between atoms. (**B**–**E**) Schematic diagrams of the molecular structures of Quercetin with NR4A1, TNFSF10, EGF, and CCL5, respectively. (**F**–**J**) Schematic diagrams of the molecular structures of Tretinoin with NR4A1, TNFSF10, CXCR4, THBD, and CCL5, respectively. (**K**, **L**) Schematic diagrams of the molecular structures of bisphenol A with NR4A1 and CXCR4, respectively. (**M**) Schematic diagram of the molecular structure of Curcumin with EGF. (**N**) Schematic diagram of the molecular structure of Valproic Acid with EGF. (**O**, **P**) Schematic diagrams of the molecular structures of Simvastatin with TNFSF10 and CX3CR1, respectively.

Molecular docking simulation techniques were used to explore the possible action mechanisms of these drugs. Due to the absence of 3D molecular structures for some compounds and ATF3, docking was only performed between 7 hub genes (excluding ATF3) and 7 compounds to evaluate the binding affinity as potential therapeutic targets. The binding energies between proteins and molecules are shown in Table 1. Binding energy information for drug molecules predicted by molecules are shown in Table 1. Binding energy information for drug molecules predicted by non-corresponding hub genes and those with unstable binding or no hydrogen bond connections between proteins and molecules were excluded. Among them, Tretinoin and Curcumin showed the highest binding energies with NR4A1 and EGF. Figure 14 presents schematic diagrams of the molecular structures of proteins and molecules.

**Table 1:** Molecular docking information.

Drug molecules	PubChem CID	Binding energy (kcal/mol)						
		NR4A1	TNFSF10	CX3CR1	EGF	THBD	CXCR4	CCL5
Perfluorooctanoic acid	9554		−1.89					
Quercetin	5,280,343	−4.44	−4.70		−4.34			−3.32
Tretinoin	444,795	−6.48	−5.34			−4.86	−5.57	−5.51
bisphenol A	6623	−5.12					−4.53	
Curcumin	969,516				−6.08			
Valproic acid	3121				−4.35			
Simvastatin	54,454		−5.06	−4.71				

## Discussion

This study studied the co-differentially expressed genes of HN and DN based on the GEO database. The network diagram was obtained by performing PPI analysis on the differential genes and imported into Cytoscape. The algorithms in Cytohubba and MCODE were used for screening to obtain eight hub genes: NR4A1, TNFSF10, CX3CR1, EGF, THBD, CXCR4, CCL5, and ATF3. In the DN dataset, all of these genes showed significant differences between the disease and control groups. However, TNFSF10 did not show an important difference between the two groups in the HN dataset. This may be due to limited sample size, individual differences, and experimental design. CX3CR1, THBD, CXCR4, and CCL5 are significantly up-regulated in HN, while NR4A1, EGF, and ATF3 are significantly down-regulated. NR4A1, EGF, THBD, CXCR4, CCL5, and ATF3 are up-regulated considerably in DN, and TNFSF10 and CX3CR1 are significantly down-regulated.

Single-cell sequencing analysis showed that TNFSF10 and NR4A1 were the most highly expressed in cells of HN and DN. Notably, NR4A1 exacerbates the pathological process of DN by activating Mff-mediated mitochondrial fission and inhibiting Parkin-mediated mitochondrial autophagy^27^. In the context of hypertension, the absence of NR4A1 aggravates renal injury. The lack of NR4A1 leads to higher proteinuria and reduced glomerular filtration rate, accompanied by a significant increase in tubular atrophy, casts, and interstitial fibrosis^28^. TNFSF10 alleviates the progression of renal fibrosis by inhibiting epithelial-mesenchymal transition in hypoxia-induced renal injury^29^. In DN, the downregulation of CX3CR1 is associated with increased inflammatory response^30^. EGF is closely related to the repair and regeneration processes of the kidney^31–33^. In addition, EGF regulates cell proliferation, migration, growth, and differentiation through its receptor (EGFR), which is crucial for the normal function of kidney cells^34^. Finally, in DN models, the activation of ATF3 is associated with pathological processes, while inhibition of ATF3 shows a protective effect. For example, using the Raf inhibitor, GW5074 can reduce the excretion of ATF3 in urine and lower serum creatinine levels, indicating that the activation of ATF3 in diabetic nephropathy may be related to disease progression^35^.

GO analysis shows these genes are mainly associated with peptide hormone, cytokine receptor binding, and chemokine receptor binding. In terms of HN, hypertension is related to changes in specific peptides in urine, and these peptides are related to vascular calcification and sodium regulation^36^. The external side of the peripheral blood membrane has a complex association with HN and DN. Diabetes and hypertension may both lead to changes in plasma components and affect the coagulation and fibrinolysis systems, thereby promoting the development of nephropathy^37,38^. Studies have shown that the binding of cytokines and chemokine receptors is closely related to the inflammatory response, and the inflammatory response is one of the causes of the development of HN and DN^39–41^. KEGG enrichment indicates Aldosterone synthesis, secretion, and the FoxO signalling pathway. In addition, KEGG enrichment also shows the AGE-RAGE signalling pathway in diabetic complications. Studies have shown that abnormally elevated aldosterone levels are closely related to hypertension and its complications^42^. The expression level of the RAGE is up-regulated in diabetic and hypertensive patients, indicating that the activation of RAGE may be one of the common pathological mechanisms of these diseases^43^. GSEA analysis reveals specific pathway enrichment through all genes in the two datasets. The HN dataset shows associations with macrophage activation^44^ and proteasome^45^. The DN dataset shows associations with chemokine activity^40^ and AGE-RAGE signalling pathway in diabetic complications^46^.

Next, immune cell infiltration analysis found that the abundance of activated natural killer cells was significantly increased in patients with HN and DN^47,48^. Activated natural killer cells can release various inflammatory factors to aggravate the inflammatory response in the kidney. In patients with HN, a significant increase in the abundance of monocytes will trigger an inflammatory response, promote renal fibrosis, affect the vasomotor function of renal blood vessels, and change renal hemodynamics^11^. In patients with DN, an increased abundance of activated dendritic cells will promote the inflammatory response and lead to immune dysregulation and renal fibrosis^49^.

Studies have shown that hsa-miR-200b-5p may affect the proliferation, apoptosis, migration, and other functions of cells by regulating the expression of specific genes in kidney cells^50^. Hsa-miR-23b-5p regulates the expression of p53 and p38MAPK and participates in the pathological process of diabetic nephropathy under high glucose conditions^51^. KCNQ1OT1 is significantly upregulated in the plasma of DN patients and in high-glucose (HG)-induced renal tubular epithelial cells (HK-2). By targeting miR-506-3p to inhibit its expression, KCNQ1OT1 leads to increased levels of reactive oxygen species (ROS) and malondialdehyde (MDA), and reduces superoxide dismutase (SOD) activity^52^. NEAT1 is significantly upregulated in plasma of DN patients and high-glucose-induced glomerular mesangial cells. By sponging miR-27b-3p to release ZEB1 (a core transcription factor of epithelial-mesenchymal transition, EMT), NEAT1 leads to accumulation of ECM proteins (such as fibronectin and collagen IV) and EMT of renal tubular epithelial cells^53^. In DN, increased acetylation of STAT3 is associated with enhanced inflammatory response in diabetic kidneys^26^; in HN, the absence of STAT3 leads to impaired cardiac systolic function and metabolic homeostasis^54^. The polymorphism of the NFKB1 gene is related to diabetes and its complications, especially the development of DN^55^. It is reported that USF1 affects the development of DN by regulating glucose-mediated expression of TGF-β1 in glomerular mesangial cells^56^. Understanding the relationship between miRNAs, TFs, and HN and DN plays an important role.

We used NetworkAnalyzer and the CTD to predict drug chemicals that may have therapeutic potential. Subsequently, molecular docking simulations were performed on the predicted chemicals to reveal potential therapeutic drugs or compounds for further investigation. Studies have shown that perfluorooctanoic acid, bisphenol A, and particulate matter are associated with an increased risk of hypertension and diabetes^57,58^. In a high-glucose environment, renal tubular epithelial cells activate an abnormal ferroptosis process, while quercetin counteracts this process by inhibiting ferroptosis and activating Nrf2 expression, showing its renal protective effect^59^. Curcumin, simvastatin, and cadmium positively treat HN and DN^60,61^. Similarly, we inferred the potential relationship between target mRNAs and specific diseases through DisGeNET and MalaCards databases. The relationship between atherosclerosis, hypertensive nephropathy, and diabetic nephropathy is mainly reflected in their common pathophysiological mechanisms^62,63^. Multiple studies have pointed out that diabetes mellitus significantly correlates with HN and DN^64^. Finally, we used the ENCORI database to identify RBPs that interact with RNA molecules, including CHTOP, EIF4E, and IGF2BP3^65–67^, which is essential in our in-depth understanding of RBP regulation of RNA molecules.

In summary, this integrative multi-omics analysis uncovers the molecular shared characteristics and potential therapeutic targets of HN and DN, laying a foundation for the development of personalized diagnosis and treatment strategies for these two diseases.

## Conclusion

This study systematically unravels the molecular commonalities and potential regulatory networks between HN and DN through integrated bioinformatics analysis and multi-dimensional data mining, providing novel theoretical insights for the collaborative diagnosis and treatment of these two diseases.

## Electronic supplementary material

Below is the link to the electronic supplementary material.

Supplementary Material 1

## Data Availability

All data generated or analyzed during this study will be available upon request to the corresponding author.
