# Supplementary material for: Identify the co-expressed genes of hypertensive nephropathy and diabetic nephropathy
Source: Sci Rep. 2025 Jun 3;15:19418. doi: 10.1038/s41598-025-04679-w (PMC12134351; doi:10.1038/s41598-025-04679-w)
Supplement: Supplementary file 1 — Supplementary Material 1 [file 41598_2025_4679_MOESM1_ESM.pdf]

Identify the Co-expressed Genes of Hypertensive Nephropathy and Diabetic Nephropathy

Dr Yanmin Wang
Institute of Statistics and Applied Mathematics, Anhui University of Finance and Economics, Bengbu, China
120081541@aufe.edu.cn
0000-0002-2802-8000
Yiming Wang*
Institute of Statistics and Applied Mathematics, Anhui University of Finance and Economics, Bengbu, China
3202300266@aufe.edu.cn
Dr Jing Jin
School of Accountancy, Anhui University of Finance and Economics, Bengbu, China
120081547@aufe.edu.cn
Dr Bing Ma*
Bengbu Third People's Hospital attached to Bengbu Medical University, Bengbu, China
15980205163@163.com

Table S1 The 42 DEGs.

DEGs name	
CX3CR1	CA2
CD248	PTPRE
MNDA	NFIL3
P2RY13	SLC22A4
CR2	AVPI1
GATM	CXCR4
TNFSF10	CCRL2
FTCD	DEFB1
ACADM	NR4A2
GIMAP6	PHLDA2
OAS1	ABP1
PLS1	EGF
BTN3A2	TRIB1
CCL5	PLK2
NR4A1	GATA6
AGTR1	LTF
TFCP2L1	ATF3
MGLL	ZNF331
SLCO2A1	HBA2
HOXB6	LRRC32
SPINK1	THBD

Table S2 The interaction relationship table of six different types of molecular complexes.

other	mRNA	node	attribute
hsa-miR-1248	CCL5	NR4A1	mRNA
hsa-miR-200b-5p	NR4A1	TNFSF10	mRNA
hsa-miR-23b-5p	CCL5	CX3CR1	mRNA
hsa-miR-3059-5p	CX3CR1	EGF	mRNA
hsa-miR-3065-3p	ATF3	THBD	mRNA
NFIL3	CX3CR1	CXCR4	mRNA
STAT3	ATF3	CCL5	mRNA
NFKB1	ATF3	ATF3	mRNA
USF1	THBD	hsa-miR-1248	miR
USF2	THBD	hsa-miR-200b-5p	miR
EGR1	ATF3	hsa-miR-23b-5p	miR
STAT3	CCL5	hsa-miR-3059-5p	miR
NFKB1	NR4A1	hsa-miR-3065-3p	miR
NFKB1	TNFSF10	NFIL3	TF
NFKB1	CXCR4	STAT3	TF
USF1	ATF3	NFKB1	TF
USF2	ATF3	USF1	TF
Cisplatin	TNFSF10	USF2	TF
Cyclosporine	NR4A1	EGR1	TF
perfluorooctanoic acid	TNFSF10	Cisplatin	drug
Quercetin	ATF3	Cyclosporine	drug
Tretinoin	TNFSF10	perfluorooctanoic acid	drug
bisphenol A	NR4A1	Quercetin	drug
Curcumin	EGF	Tretinoin	drug
Valproic Acid	TNFSF10	bisphenol A	drug
Particulate Matter	THBD	Curcumin	drug
Simvastatin	ATF3	Valproic Acid	drug
Cadmium	ATF3	Particulate Matter	drug
Cisplatin	ATF3	Simvastatin	drug
Cisplatin	EGF	Cadmium	drug
Cisplatin	CXCR4	Atherosclerosis	disease
Cisplatin	NR4A1	Glioblastoma	disease
Cyclosporine	TNFSF10	Pulmonary Fibrosis	disease
Cyclosporine	ATF3	Asthma	disease
Cyclosporine	EGF	Hepatitis B	disease
perfluorooctanoic acid	ATF3	Hepatitis C	disease
Quercetin	CCL5	Diabetes Mellitus	disease
Quercetin	EGF	CHTOP	RBP
Quercetin	NR4A1	EIF4E	RBP
Quercetin	TNFSF10	HNRNPK	RBP
Tretinoin	ATF3	IGF2BP3	RBP
Tretinoin	CCL5	YTHDF3	RBP

Tretinoin	EGF	HNRNPA2B1	RBP
Tretinoin	CXCR4	RBM47	RBP
Tretinoin	THBD	YBX1	RBP
Tretinoin	NR4A1	RBFOX2	RBP
bisphenol A	ATF3	RBM10	RBP
bisphenol A	CXCR4		
Curcumin	CXCR4		
Curcumin	TNFSF10		
Curcumin	ATF3		
Valproic Acid	ATF3		
Valproic Acid	EGF		
Valproic Acid	CXCR4		
Particulate Matter	NR4A1		
Particulate Matter	TNFSF10		
Particulate Matter	ATF3		
Particulate Matter	CCL5		
Simvastatin	CCL5		
Simvastatin	THBD		
Simvastatin	CX3CR1		
Simvastatin	TNFSF10		
Cadmium	EGF		
Cadmium	CXCR4		
Cadmium	NR4A1		
Cadmium	CX3CR1		
Cadmium	TNFSF10		
Atherosclerosis	NR4A1		
Glioblastoma	TNFSF10		
Pulmonary Fibrosis	EGF		
Asthma	CCL5		
Hepatitis B	CCL5		
Hepatitis C	CCL5		
Diabetes Mellitus	CCL5		
Glioblastoma	EGF		
Pulmonary Fibrosis	CCL5		
Asthma	CX3CR1		
Asthma	THBD		
Diabetes Mellitus	CX3CR1		
Diabetes Mellitus	THBD		
CHTOP	NR4A1		
EIF4E	ATF3		
HNRNPK	NR4A1		
IGF2BP3	NR4A1		
YTHDF3	CXCR4		
HNRNPA2B1	TNFSF10		

RBM47	ATF3
YBX1	THBD
RBFOX2	ATF3
RBM10	ATF3
CHTOP	ATF3
EIF4E	NR4A1
IGF2BP3	EGF
IGF2BP3	CXCR4
IGF2BP3	ATF3
YTHDF3	ATF3
YTHDF3	NR4A1
HNRNPA2B1	THBD
HNRNPA2B1	ATF3
RBM47	THBD
